# STING activation by teniposide: a potential direct mechanism beyond cGAS stimulation

**DOI:** 10.3389/fimmu.2025.1677836

**Published:** 2026-01-02

**Authors:** Javier Arranz-Herrero, Laura Marquez-Cantudo, Sergio Rius-Rocabert, Rubén M. Buey, Adrian Velazquez-Campoy, Vicent Tur-Planells, Adolfo Garcia-Sastre, Lisa Miorin, Beatriz de Pascual-Teresa, Claire Coderch, Estanislao Nistal-Villan

**Affiliations:** 1 Microbiology Section, Departamento de Ciencias, Farmacéuticas y de la Salud, Facultad de Farmacia, Universidad San Pablo-CEU, CEU Universities, Madrid, Spain; 2 Transplant Immunology Unit, National Center of Microbiology, Instituto de Salud Carlos III, Madrid, Spain; 3 Institute of Applied Molecular Medicine (IMMA), Department of Basic Medical Sciences, Facultad de Medicina, Universidad San Pablo-CEU, CEU Universities, Madrid, Spain; 4 Departamento de Química y Bioquímica, Facultad de Farmacia, Universidad San Pablo-CEU, CEU Universities, Madrid, Spain; 5 Metabolic Engineering Group - Unit Associated to CSIC through IRNASA, Department of Microbiology and Genetics, Universidad de Salamanca, Salamanca, Spain; 6 Institute of Biocomputation and Physics of Complex Systems (BIFI), Universidad de Zaragoza, Zaragoza, Spain; 7 Instituto de Investigación Sanitaria Aragón (IIS Aragón), Zaragoza, Spain; 8 Centro de Investigación Biomédica en Red en el Área Temática de Enfermedades Hepáticas y Digestivas (CIBERehd), Madrid, Spain; 9 Departamento de Bioquímica y Biología Molecular y Celular, Universidad de Zaragoza, Zaragoza, Spain; 10 Department of Microbiology, Icahn School of Medicine at Mount Sinai, New York, NY, United States; 11 Global Health and Emerging Pathogens Institute, Icahn School of Medicine at Mount Sinai, New York, NY, United States; 12 Department of Medicine, Division of Infectious Diseases, Icahn School of Medicine at Mount Sinai, New York, NY, United States; 13 The Tisch Cancer Institute, Icahn School of Medicine at Mount Sinai, New York, NY, United States; 14 Department of Pathology, Molecular and Cell-Based Medicine, Icahn School of Medicine at Mount Sinai, New York, NY, United States; 15 The Icahn Genomics Institute, Icahn School of Medicine at Mount Sinai, New York, NY, United States

**Keywords:** STING, Teniposide, IFN-β, Isothermal titration calorimetry (ITC), High-throughput virtual screening (HTVS), Molecular docking, Cyclic dinucleotides (CDNs), Immunotherapy drug repurposing

## Abstract

**Introduction:**

The STimulator of Interferon Genes (STING) is a key adaptor protein in the innate immune response to cytosolic DNA, making it a promising therapeutic target. Identifying novel STING ligands could provide new opportunities for immune modulation.

**Methods:**

We employed high-throughput virtual screening to identify potential STING ligands and selected Teniposide, an anticancer drug primarily used for infant leukemia. Direct binding of Teniposide to STING’s cytosolic domain was confirmed via isothermal titration calorimetry (ITC) and validated using a double mutant STING variant unable to bind Teniposide. Computational docking and molecular dynamics simulations were performed to characterize the binding mode.

**Results:**

Teniposide activated the IFN-β signaling pathway in a STING-dependent manner, independent of dsDNA sensors cyclic GMP-AMP synthase (cGAS) and Interferon Gamma Inducible Protein 16 (IFI16). ITC confirmed direct interaction, and the STING double mutant abolished binding. Computational analyses revealed a symmetrical binding mode involving two Teniposide molecules interacting with STING.

**Discussion:**

These findings suggest that Teniposide activates STING through a previously unrecognized, cGAS-independent mechanism, while retaining potential for canonical cGAS-STING stimulation. Our combined computational and experimental evidence supports repurposing Teniposide as a STING agonist, highlighting new therapeutic possibilities for innate immune stimulation.

## Introduction

STING is an intracellular protein that plays a crucial role in innate immune response initiation in vertebrates. Serving as an adaptor protein, it becomes activated through various mechanisms, including double-stranded DNA (dsDNA) from both endogenous and exogenous sources such as pathogens (1, 2). The stimulation of STING and its subsequent role in the activation of innate and adaptive immune responses have garnered considerable attention in cancer immunotherapy due to its therapeutic potential (3–6).

Activation of STING by natural small molecule ligands involves the dsDNA sensor protein cyclic GMP-AMP synthetase (cGAS), leading to the generation of the secondary metabolite cyclic GMP-AMP (cGAMP) (7, 8). Moreover, STING signaling can be triggered by cyclic di-nucleotides (CDNs) of bacterial origin, such as cdiGMPs, cdiAMPs, or cdiGAMPs (9, 10). In addition to CDNs, STING can be stimulated through protein-protein interaction mechanisms (11). Notably, the interaction with IFI16 holds particular significance. Consequently, therapeutic strategies targeting STING have predominantly focused on the development of molecules capable of direct binding or modulating STING upstream activation.

Current STING cyclic dinucleotides ligands fall into two main categories: natural ligands, represented by CDNs, and small organic non-CDNs. CDNs include the natural ones (cGAMP, cdiGMP and cdiAMP) and the modified cyclic di-nucleotides (mCDNs). Notably, mCDNs typically aim to counter CDN degradation by ENPP1 (12), while non-CDNs prioritize the identification of small molecules capable of binding to the CDN binding pocket in STING, mimicking the conformational changes triggered by cGAMP (11). Some of these ligands are currently advancing through clinical trials for different purposes, including cancer treatment (https://www.clinicaltrials.gov codes NCT03843359, NCT06626633, and NCT06601296).

High Throughput Virtual Screening (HTVS) of drug libraries is a compelling approach for identifying small molecule STING ligands. In this study, we conducted HTVS of NIH libraries to pinpoint potential STING-binding candidates. Surprisingly, Teniposide, a well-established Topoisomerase II inhibitor (13), emerged as a potential small molecule STING ligand. Subsequent functional and biochemical validation of Teniposide binding to STING suggests its potential utility in triggering STING activation.

## Materials and methods

### High throughput virtual screening

#### Protein selection and preparation

At the beginning of this project only the soluble domain of STING was available at the Protein Data Bank (PDB). For the initial calculations we selected two wild type PDB high resolution crystal structures of the open (PDB code 4F5W), and closed (PDB code 4LOI) conformations (14, 15). The selected human proteins were superimposed and prepared using the Protein Preparation Wizard (Schrödinger Release 2024-1: Protein Preparation Wizard; Epik, Schrödinger, LLC, New York, NY, 2024; Impact, Schrödinger, LLC, New York, NY; Prime, Schrödinger, LLC, New York, NY, 2024). The docking was delimited by a 15 Å-size box located in the centroid between R238 of both subunits of PDB code 4LOI.

#### Library preparation and screening protocol

The libraries selected for the virtual screening were the NIH (National Institutes of Health) (https://www.nih.gov/) NCI databases: Natural Set 3, Natural Set 4, Natural Set 5, Diversity Set 3, Diversity Set 4 and Diversity Set 5.

A ligand-flexible docking was performed with the selected libraries using the HTVS Glide module (Schrödinger Release 2024-1: Glide, Schrödinger, LLC, New York, NY, 2024). Ligand duplicates were eliminated; however, no previous filtering was carried out. Sequential dockings were performed with increasing precision on both prepared protein structures. The results were evaluated based on the predicted ligand-receptor interaction energies expressed in kcal·mol^-1^ (**Supplementary Figure 1**).

### Bone-marrow-derived macrophage cultures

C57BL/6 mice were used for the extraction of the bone marrow and the obtention of hematopoietic cells from the tibia and femur.

C57BL/6J mice were obtained from The Jackson Laboratory (Bar Harbor, ME) and housed under specific pathogen-free conditions in compliance with institutional animal care and ethics committee guidelines as part of procedures approved under protocol IACUC-2013–1408 by the Institutional Animal Care and Use Committee of the Icahn School of Medicine at Mount Sinai (ISMMS). Bone marrow-derived macrophages (BMDMs) were isolated from the femurs of sacrificed corpses from control animals otherwise discarded. Experimental groups included equal numbers of male and female mice.

In the extraction process 5mL needles (Soft-Ject) were used, 25G 5/8” needles (BD MicrolanceTM 3), 21G 5/8” needles (Terumo) and PBS 1X (Gibco; [+] CaCl_2_, [+] MgCl_2_). Macrophages are conserved in complete RPMI medium (Gibco RPMI Medium 1640 (1X) + GlutaMAX™-L, supplemented with fetal bovine serum (FBS) 10%, Hepes 125mM, Sodic Pyruvate 1mM, Beta-Mercaptoethanol 100µM and Penicillin/Streptomycin). In addition, cells receive a treatment with 25 ng/mL of GM-CSF differentiating cytokine (Peprotech 130-095-372). We assume that this treatment can stimulate the differentiation of bone marrow monocytes into M1-like derived macrophages (BMDM). Alternative differentiation protocols could generate more defined macrophages phenotypes. Cells were incubated in 24 or 12-well plates (BD FalconTM) with a population density of 5x10^5^ cells per well. The incubations were carried out in the “Thermo Scientific Steri-Cult” incubator at 37°C, 5% of CO_2_ and 90% of humidity. After 72h, the cell media was changed for another three days. For the washing steps, PBS 1X (Gibco; [+] CaCl_2_, [+] MgCl_2_) is used.

### THP1 cultures

Thp1 ATCC^®^ TIB-202™ cell cultures are human monocyte cells obtained from peripheral blood of patients with acute monocytic leukemia. Thp1 cells are conserved in complete RPMI medium (Gibco RPMI Medium 1640 (1X) + GlutaMAXTM-L, supplemented with FBS 10%, Hepes 125mM, Sodic Pyruvate 1mM, β-Mercaptoethanol 100µM and Penicillin/Streptomycin.

Cell lines were incubated in a 10mL flask during the experiments. The incubations were carried out in the “Thermo Scientific Steri-Cult” incubator at 37°C, 5% of CO_2_ and 90% of humidity.

### Generation of THP-1 CRISPR/Cas9 knockouts

THP-1 STING, cGAS and IFI16 knockout cell lines were generated using the CRISPR-CAS9 sgRNA guides, Hs.Cas9.TMEM173.1.AA (GCTGGGACTGCTGTTAAACG), Hs.Cas9. MB21D1.AB (ACGAAGCCAAGACCTCCGCC), Hs.Cas9.IFI16.1.AM (TCAGCCAGGTCTTCAAGCGT), respectively (IDT, Coralville, IA, USA) and the SG Cell Line 4D-Nucleofector™ X Kit S (Lonza, Basel, Switzerland) in a 4D-nucleofector (Lonza, Basel, Switzerland) following the manufacturer’s instructions. 72 hours after treatment, a western blot was performed for the desired proteins and better candidates were single cell plated for obtaining complete knockout clones.

### *In vitro* experiments

Macrophages, THP1 cells, or THP1 KO cells for cGAS, IFI16, or STING were treated with cGAMP or Teniposide at different time points or concentrations in each experiment. The cells were disrupted by adding 0.5mL of Tri-Reagent solution (Sigma). After that, the cells are transferred into eppendorf tubes with 0.1mL of chloroform. All the Eppendorf tubes were mixed softly for 15s. After 2–15 min at room temperature, the samples were centrifuged at 12000G for 15 min at 4°C. The aqueous phase was transferred into a new eppendorf with 250µl of isopropanol. After mixing and waiting 5 to 10 minutes at room temperature, the samples were again centrifuged at maximum speed for 10 minutes at 4°C. The supernatant was discarded, and the pellet was resuspended in 0.5mL of ethanol 75% and centrifuged again at maximum speed for 5 min at 4°C. Again, the supernatant was discarded, and the tubes were dried out. Finally, the pellet was resuspended in 20-30µL of distilled water. Subsequently, a DNA digestion protocol was carried out using RNAse-free DNAse kit (QIAGEN). For cDNA synthesis, the High-Capacity cDNA Reverse Transcription Kit (Applied Biosystems) was used according to the manufacturer’s instructions in a Verity^®^ thermocycler (37°C for 120 minutes - 85°C for 10 minutes - 96°C for 1 minute). Relative expression was measured by qPCR using SYBR green. The list of primers utilized is detailed in **Supplementary Table S1**.

#### Western blot (WB)

Generic WB utilizing specific antibodies was used to detect TBK1 (Cell signaling Ref: D1B4), phosphorylated TBK1 (Cell signaling ref: D52C2), IRF3 (Cell signaling Ref: D83B9), phosphorylated IRF3 Cell Signaling ref: 4d4g), β-actin (Cell signaling ref: 4967), STING (Cell signaling ref: D2P2F), cGAS (Cell signaling ref: D1D3G), and IFI16 (Cell signaling ref: D8B5T) proteins. Proteins were separated by SDS-PAGE (Biorad, Hercules, CA) and then transferred into PVDF membranes (Millipore, Burlington, MA), following the manufacturer´s instructions.

### Recombinant STING-LBD expression

The cDNA of hSTING lacking 138 N-terminal residues was PCR amplified and inserted into an in-house modified pET15b bacterial expression plasmid (16). Missense mutations were introduced by site-directed mutagenesis using the QuikChange II method (Agilent Technologies). All DNA constructs were corroborated by DNA sequencing. Recombinant proteins were expressed in *Escherichia coli* strain BL21 (DE3) and purified by nickel-chelating affinity chromatography according to standard protocols. The His_8_ tag present at the N terminus of the fusion proteins was cleaved by digestion with tobacco etch virus protease (His_6_-tagged), and the protease, together with residual uncleaved protein, was removed by a second nickel-affinity chromatography. The cleaved protein was then injected into a HiPrep Sephacryl S-300 16/60 HR size-exclusion chromatography column (Cytiva Life Sciences) previously equilibrated in buffer 20 mM Tris-HCl, 300 mM KCl, 1 mM DTT (Dithiothreitol), pH 8.0. Fractions containing recombinant proteins were pooled, concentrated in a 10 kDa cutoff Amicon Ultra Centrifugal Filter (Millipore), flash-frozen into liquid nitrogen and stored at -80°C until used.

### Isothermal titration calorimetry

ITC experiments were conducted using an Auto-iTC200 (MicroCal, Malvern Panalytical). Recombinant STING (20 µM) in 20 mM HEPES buffer (150 mM KCl, pH 8.0) was titrated with 300 µM solutions of Teniposide, α-mangostin, or 2´3´-cGAMP at 25°C. The heat released during binding was recorded, integrated, and analyzed using a single binding site model in the Origin software package (OriginLab Corporation).

### Modeling of the complex of STING with two units of Teniposide

#### Molecular dynamics simulation of the interaction of two Teniposide units

The Teniposide structure was quantum mechanically parametrized by performing the geometry optimization and charge distribution at the RHF/6-31G** level of theory using Hartree-Fock with Gaussian 03 (Gaussian, Inc., Wallingford, CT, 2004), Revision C.02. available at https://gaussian.com/glossary/g03/). The RESP (*Restrained Electrostatic Potential*) charges were obtained using the Antechamber package included in Amber Tools module of AMBER16 (http://ambermd.org/). The bonded and nonbonded parameters were assigned with the general AMBER forcefield (GAFF) of the AMBER General Force Field for organic molecules (Version 1.7, November 2013).

Two unbound Teniposide units were simulated in explicit water to account for the possible interactions that could allow them to interact at the same time with the STING homodimer. The starting point for the molecular dynamics (MD) simulation was obtained by manually separating the two Teniposide molecules by 10 Å. This initial conformation was immersed in an approximately 5700 TIP3P water molecule octahedron. The sander and *pmemd* modules of AMBER16 were used for the restrained and unrestrained MD simulations (17). The electrostatic interactions were treated with the smooth particle mesh Ewald method with a grid spacing of 1Å while applying periodic boundary conditions (18–20). The non-bonded interactions were treated with a cutoff of 9 Å, the SHAKE algorithm was applied to all bonds and an integration step of 2.0 fs was used throughout the simulation (21). After the preceptive initial minimization, the system was heated to 300K in 25 ps where the solute was maintained in its initial position by positional restraints of 50 kcal mol^-1^ Å^-1^. The solvent was then allowed to redistribute in the equilibration phase of 1 ns where the solute was slowly released to explore the mutual adaptation of the water molecules to the solute. The system was simulated for 100 ns after which the resulting trajectory was analyzed with the *ptraj* module of AMBER16 to run a cluster analysis and extract the most populated 10 conformers depicting the interactions of the two Teniposide molecules. A single point energy of the major conformers in vacuum was calculated, then the relative energies of each conformer were extracted and used to calculate the probability of the microstates (%) as defined by the Boltzmann distribution formula to account for the probability of the microstates:


$$Pi=ne^{-\frac{Ei}{RT}}/{\sum_{j}^{n}e}^{-\frac{Ei}{RT}}$$

In this Boltzman distribution equation *n* is the number of structures that make up each of the conformer clusters (*i*), *Ei* is the energy of the average structure of each cluster, *T* is the temperature expressed in *kelvin*, *R* is the gas constant (1.987 10–^3^ kcal·mol^-1^·K^-1^) and *j* is the total number of clusters extracted from the MD simulation.

#### Docking of the two tethered Teniposide units

In order to be able to carry out the docking of the representative structure of the extracted clusters from the previous MD simulation, the Teniposide molecules of the three most populated clusters (co, c1 and c2) were tethered by alkyl linkers (each at strategic positions (**Supplementary Figure S2**)) to avoid any possible conformational change and to be able to treat them as one single molecule. These alkyl linkers were added in positions that would not interfere in the ligand-protein interactions. The tethered Teniposides were prepared with the Maestro *LigPrep* module of the Schrödinger suite (Schrödinger Release 2023-2: LigPrep, Schrödinger, LLC, New York, NY, 2023.).

The selected proteins for the docking were PDB codes 4LOI for the closed conformation (15); PDB codes 6DXL and 6DXG for the semi-open conformations (22); and PDB code 6MXE for the open conformation (23). The proteins were all superimposed to the coordinates of 6MXE and prepared using the Protein Preparation Wizard of the Schrödinger suite (24). No missing side chains were added and the protonation state of titrable groups was calculated at pH 7. The docking was delimited by a box centered at a centroid between Y261 of the two monomers of 6MXE, that measured either 30 Å for the closed and semi open conformation, and 35 Å for the open conformation (Schrödinger Release 2024-1: Glide, Schrödinger, LLC, New York, NY, 2024.). The docking calculations were carried out using the standard precision (SP) mode of the Glide Schrödinger suit (Schrödinger Release 2024-1: Glide, Schrödinger, LLC, New York, NY, 2024.).

#### Molecular dynamic simulations of the complex of STING with two untethered Teniposide units

In order to build the ligand-protein complex, the most favorable docking result obtained for the tethered cluster c2 in complex to 6MXE STING structure, was superimposed to 8A2I with the missing I235 modeled in chain B. The superimposition was carried out by superimposing the 6MXE monomer that interacted the most with the tethered cluster with chain B of 8A2I as the interaction of the tethered cluster could be expected to affect the conformation of the same amino acid positions in the β-sheet lid. In addition, given that the ITC experiments were carried out with the STING R232H variant that mutation was introduced in the 8A2I structure using the mutagenesis tool provided in PyMOL (Schrödinger, L., & DeLano, W. (2020). PyMOL. Retrieved from http://www.pymol.org/pymol). The positions of the tethered Teniposide were then transferred to the 8A2I structure and to carry out the MD simulation, the two Teniposide molecules were untethered, and the parameters previously described were applied to each of the molecules. The steric clashes that the resulting complex presented were minimized by two successive rounds of minimizations in vacuum. The first round was a 50000-cycle minimization of the amino acids that interacted with the two untethered Teniposide molecules, the first 50 cycles were of steepest descent and the rest of conjugated gradient. The result of this first round was then submitted to an unrestrained minimization of 10000 cycles of which the first 5000 were of steepest descent.

The resulting optimized complex was immersed in an approximately 21500 TIP3P water molecule octahedron in the presence of approximately 0.15 M of NaCl that accounts for 59 Na^+^ ions and 47 Cl^-^ ions to achieve electroneutrality of the system. The sander and *pmemd* modules of AMBER16 were used for the restrained and unrestrained MD simulations (17). The electrostatic interactions were treated with the smooth particle mesh Ewald method with a grid spacing of 1 Å while applying periodic boundary conditions (19, 20). The non-bonded interactions were treated with a cutoff of 9 Å, SHAKE algorithm applied to all bonds and an integration step of 2.0 fs was used throughout the simulation (21). The initial solvated system was minimized and subsequently heated to 300K in 25 ps where the solute was maintained in its initial position by positional restraints of 50 kcal mol-1. The solvent was then allowed to redistribute in the equilibration phase of 1 ns where the solute was slowly released to explore the mutual adaptation of the water molecules to the solute. The simulation was extended to 400 ns to explore the adaptation of the ligands to the homodimers and snapshots of the trajectory were extracted every 20 ps. The results were analyzed with the *ptraj* module of AMBER16 to extract the Root Mean Square Deviations (RMSD) of the Teniposide molecules and the STING monomers; and the cluster analysis of the two Teniposides bound to the STING homodimer to extract the most populated binding mode. The per-residue energy decomposition analysis was carried out using the MM-ISMSA program using the whole trajectory with an offset of 2 (25).

### Statistical analysis

Statistical analysis of gene expression was calculated using Prism software. After normalization of the data, Two-way ANOVA and multiple t Student test were used to see significant differences between conditions (p-value). Statistical significance is indicated by *p ≤ 0.05, **p ≤ 0.01, ***p ≤0.005, **** p ≤ 0.001. Statistical analyses were conducted using GraphPad Prism v9.1 software.

## Results

### HTVS of NIH libraries and Teniposide as a potential STING activator

**Figure 1:**
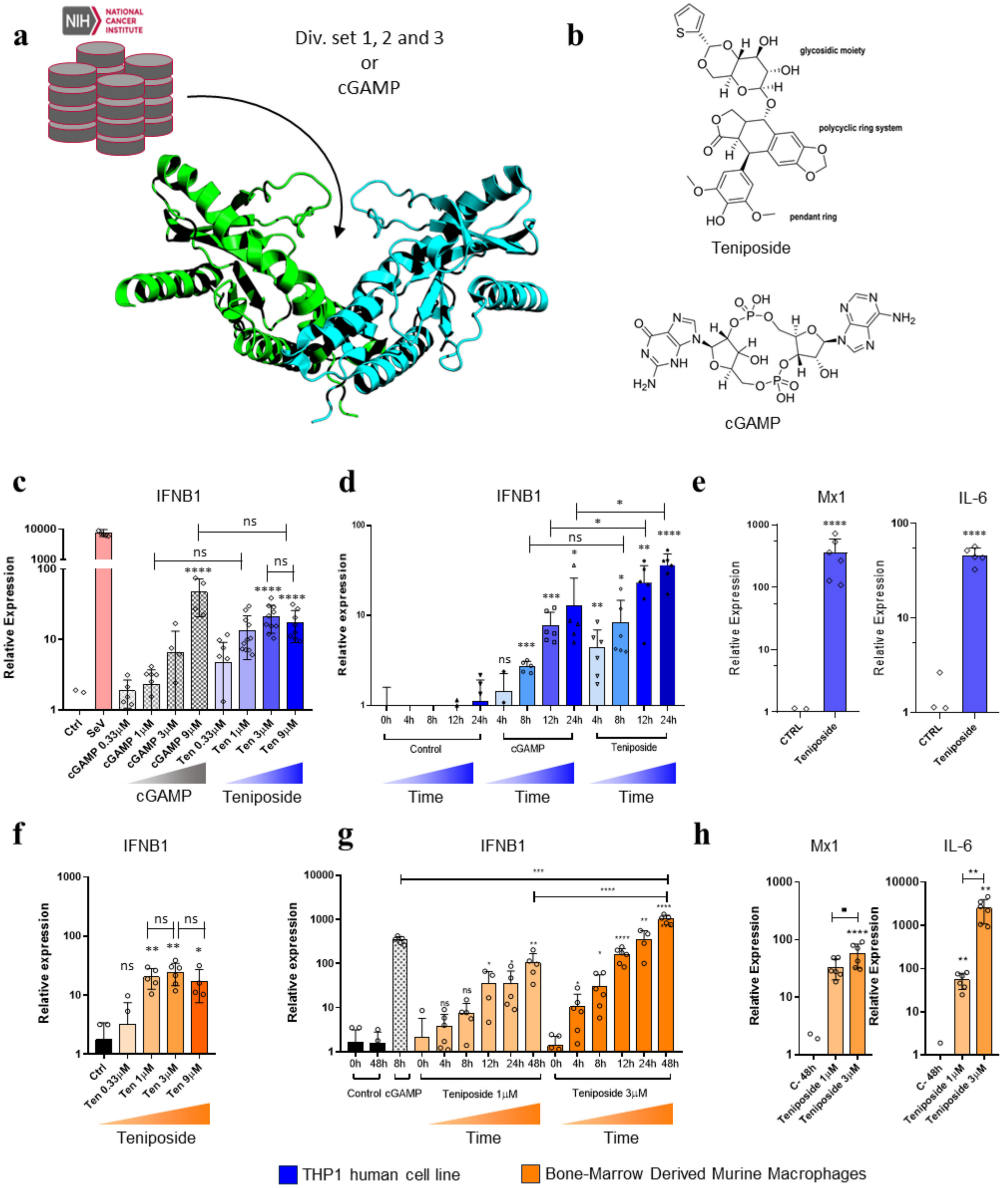
Teniposide is a potential STING agonist that induces *IFNB1* gene expression. **(a)** Schematic representation of high-throughput virtual screening HTVS of NIH libraries targeting the STING binding pocket. **(b)** Representation of the 2D structures of Teniposide and cGAMP. **(c)** Relative *IFNB1* gene expression in THP1 cells treated with varying doses of Teniposide or cGAMP. **(d)** Time-course *IFNB1* gene expression in THP1 cells treatment with cGAMP or Teniposide. **(e)***MX1* and *IL6* gene expression in THP1 cells 12h post-Teniposide treatment. **(f)** Dose response of Teniposide in BMDMs, showing relative *IFNB1* expression 24h post-treatment. **(g)***IFNB1* time-course expression in BMDM upon stimulation with Teniposide (1µM or 3µM). cGAMP induction at 8h was used as a reference control. **(h)** Relative *MX1* and *IL6* gene expression in BMDM 48h after Teniposide treatment. Statistical significance is indicated by *p ≤ 0.05, **p ≤ 0.01, ***p ≤0.005, **** p ≤ 0.001.

We carried out the HTVS of the Diversity Set libraries at the NCI (https://wiki.nci.nih.gov/display/ncidtpdata/compound+sets) using two conformations of the cytosolic domain of STING as found in PDB codes 4F5W and 4LOI for the apo open and cGAMP-bound closed conformations respectively while using cGAMP as a control ligand (**Figures 1a, b** (bottom)).

The calculations yielded a list of potential candidates that bound the CDN-binding site. Among the top-ranked molecules binding the open conformation of STING was the well-known Topoisomerase II inhibitor, Teniposide (**Figure 1b** - top) (13). Upon analysis of the binding mode, Teniposide revealed significantly higher binding energy than cGAMP (-7.047 kcal/mol vs. -5.168 kcal/mol) (**Figure 1b** - bottom) (**Supplementary Figure S1**). However, these differences could be attributed to the fact that the open conformation of STING did not establish the same interactions with cGAMP as the closed one and that Teniposide could establish more unspecific contacts.

To validate this predicted interaction in tissue culture, we conducted a series of experiments to assess the potential of Teniposide to activate STING and initiate the downstream signaling cascade, leading to IFN-β induction. We measured *IFNB1* gene expression in the human monocytic cell line (THP-1) and murine bone marrow-derived macrophages (BMDM). In all experiments, β-Actin gene *ACTB* expression served as a housekeeping gene to calculate the relative expression of the target genes, with data normalized against the negative control (DMSO), which was used as the unstimulated mock reference. Natural ligands, such as cGAMP or cyclic di-GMP, were included as positive controls.

The ability of Teniposide to induce *IFNB1* gene expression in THP-1 cells was initially assessed by stimulating the cells with increasing concentrations of the agonist cGAMP or Teniposide (**Figure 1c**). This treatment led to a significant increase in *IFNB1* gene expression at 8h post-treatment, with Teniposide showing activity at concentrations as low as 0.33 µM and reaching saturation around 3 µM. In comparison, cGAMP also stimulated cells at 0.33 µM, with further increases in concentration resulting in continued upregulation of *IFNB1* gene expression. Sendai virus (SeV), a well-established inducer of the IFN-β induction pathway, was used as a positive control. The ability to induce the *IFNB1* overtime was also tested by treating THP1 cells with 1µM of cGAMP or Teniposide (**Figure 1d**), presenting a significant gene induction as fast as 4h after treatment. Teniposide can also significantly increase the *MX1* and *IL6* gene expression (**Figure 1e**), indicating a potential activation of Type I signaling cascade and also the activation of NF-κB signaling.

To confirm that this expression was not exclusive to THP-1 cells, BMDMs were also treated with 0.33 µM, 1 µM, 3 µM, or 9 µM of Teniposide for 24 hours (**Figure 1f**). In this tissue culture model, *IFNB1* gene expression was triggered with concentrations as low as 1 µM of Teniposide. Teniposide treatment showed some level of toxicity in BMDM cultures after 24 hours, which may increase with longer exposure (data not shown). To optimize conditions for studying Teniposide kinetics, an *IFNB1* gene expression time course was performed in BMDMs using two Teniposide concentrations, 1 µM and 3 µM, with cGAMP induction at 8 hours as a reference control. Treatment with 3 µM Teniposide significantly induces *IFNB1* expression as early as 4 hours post-treatment (**Figure 1g**). The Type I IFN signaling cascade was further analyzed by determining *MX1* gene expression 12 hours after treatment (**Figure 1h**). The proinflammatory stimulation was also verified by quantifying *IL6* gene expression upon 1 µM and 3 µM Teniposide treatment.

### Teniposide-induced expression of the *IFNB1* gene is dependent on STING and may occur independently of cGAS and IFI16

**Figure 2:**
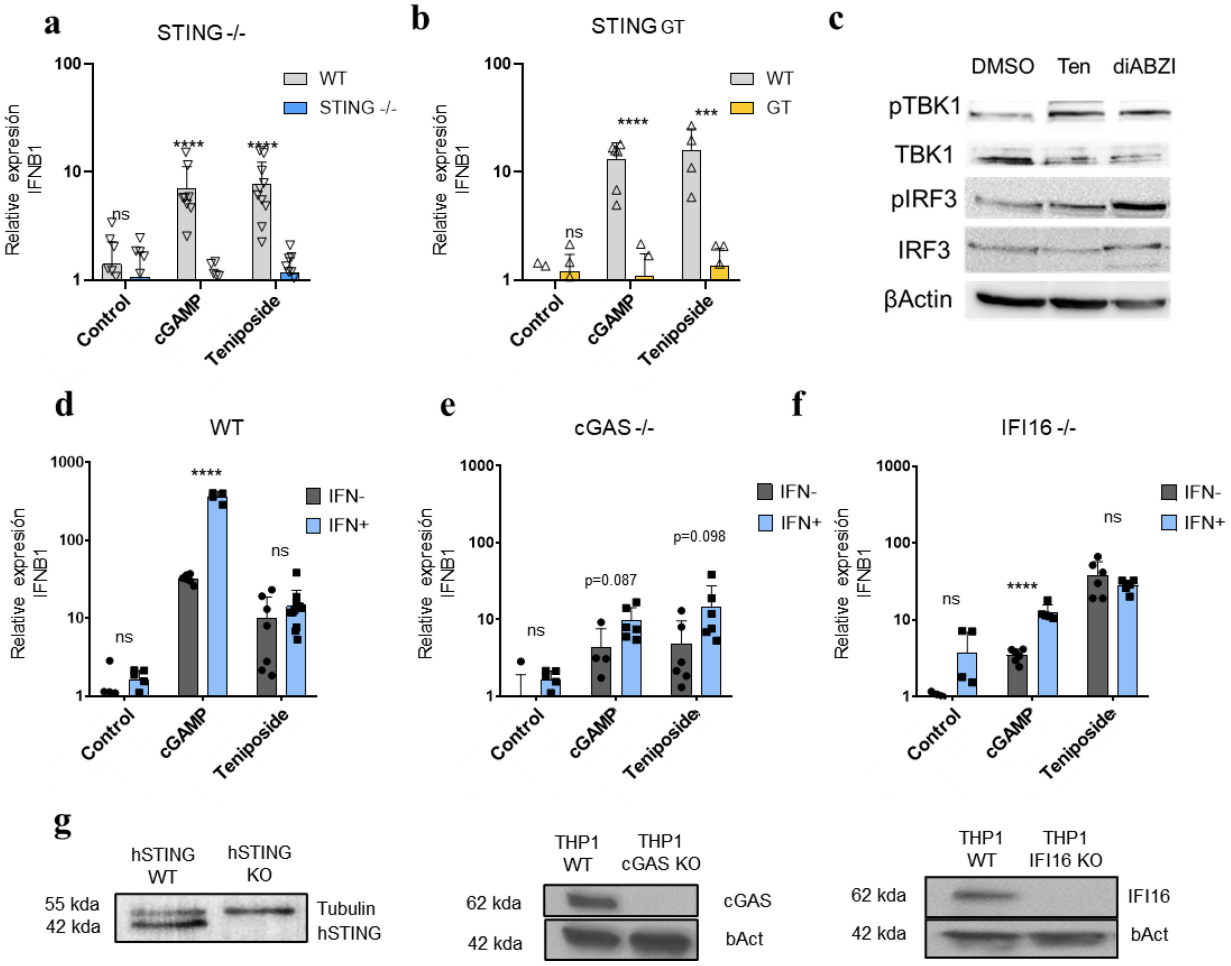
Teniposide-induced *IFNB1* expression requires STING and may occur independently of cGAS and IFI16. **(a)***IFNB1* relative gene induction measured by RT-qPCR in WT or STING KO THP1 cells after Teniposide 3µM treatment for 8h. **(b)***IFNB1* relative expression in WT or GT STING BMDM treated with 3µM Teniposide for 8h. **(c)** Western blot representing pTBK1, total TBK1, pIRF3, total IRF3 and bacting in THP1cells mock treated, treated for 4h with 3µM of Teniposide or 1µM of diABZI. **(d)***IFNB1* relative gene expression in WT THP1 cells stimulated for 18h with 10 IU of IFN-β/ml or mock-treated followed by a treatment with DMSO, cGAMP or Teniposide (3µM). **(e)** Same as in **(d)** but in WT and cGAS KO THP1 cells. **(f)** Same as in **(d)** but in WT and IFI16 THP1 KO cells. **(g)** Western blot verifying STING, cGAS or IFI16 in WT but not STING, cGAS, and IFI16 KO THP1 cells. Tubulin or β-Actin is shown as a loading control. Statistical significance is indicated by *p ≤ 0.05, **p ≤ 0.01, ***p ≤0.005, **** p ≤ 0.001.

To investigate the mechanisms underlying the activation of Type I IFN by Teniposide, the role of STING in downstream signaling and *IFNB1* expression was assessed. For this purpose, the effects of 3 µM Teniposide treatment were compared in STING-competent THP-1 cells and THP-1 cells with a STING knockout (KO). In STING-KO THP-1 cells, Teniposide failed to induce *IFNB1* expression compared to wild-type STING-competent cells, indicating that STING is an essential factor (**Figure 2a**). The STING Goldenticket (GT) isoform, which contains the I199N missense mutation, is unresponsive to CDNs. This isoform is expressed in the Tmem173gt. BMDMs from Tmem173gt mice were used to test the ability of Teniposide to stimulate *IFNB1* expression (**Figure 2b**). In this case, Teniposide treatment failed to induce *IFNB1*. cGAMP was used as a control to ensure that STING was not functional in these cells.

To assess mechanistic induction of *IFNB1* pathway downstream STING, phosphorylation of TBK1 (Ser172) and IRF3 (Ser396) was analyzed by western blot in THP−1 cells following mock treatment, teniposide (3 µM), or STING synthetic agonist diABZI (1 µM) for 4 h. Total TBK1 and IRF3 served as protein controls, with β−actin as loading control. Both teniposide and diABZI induced increased phosphorylation relative to mock treatment, consistent with pathway activation (**Figure 2c**).

Teniposide has been reported to activate the cGAS-STING pathway through a canonical mechanism involving the production of cGAMP molecules, mediated by cGAS in response to self-DNA breaks caused by the inhibition of Topoisomerase II brought about by Teniposide (26). Additionally, IFI16 can also mediate STING activation upon detecting free DNA (27). These studies suggest that Teniposide activates the cGAS-STING pathway by inducing DNA breaks, which are recognized by cGAS, leading to cGAMP synthesis and STING activation. However, the mechanism proposed here suggests that Teniposide may directly interact with STING, potentially resulting in faster STING activation. This could shorten the time between Teniposide treatment and *IFNB1* expression. To reduce the likelihood of DNA breaks and cGAS or IFI16 activation, *IFNB1* expression was analyzed 8 h post-treatment (hpt), an early time point already shown to present this induction (**Figure 1h**). To investigate the role of cGAS and IFI16 in Teniposide-induced *IFNB1* expression, WT THP1 response to Teniposide was compared with cGAS-KO and IFI16-KO THP-1 cell lines.

At 4 hpt, both cGAMP and Teniposide induced low levels of *IFNB1* expression. To address this, cells were pretreated with IFN-β or mock stimulation (control) for 18 h, in an attempt to increase the sensitivity of the IFN-β induction pathway. After pretreatment, the cells were washed and treated with cGAMP, Teniposide, or mock-treated. In this experiment, WT, cGAS KO, and IFI16 KO cells were treated with 3 µM Teniposide for 8 h, and *IFNB1* expression was measured by RT-qPCR.

Teniposide can stimulate *IFNB1* expression in cGAS and IFI16 KO BMDM (**Figures 2e, f**). Specific gene expression knockouts in the different cell lines were verified by WB and the detection of STING, cGAS, or IFI16 proteins in the corresponding KO or Wt THP1 cell respectively (**Figure 2g**).

### Teniposide directly interacts with STING *in vitro*

To investigate whether Teniposide directly binds to STING, we performed isothermal titration calorimetry (ITC), which measures binding affinities and provides insights into ligand stoichiometry and thermodynamics.

Due to the solubility challenges of full-length STING, a membrane-associated protein prone to aggregation, we used a STING-ligand binding domain (LBD) truncated version, STING-LBD (lacking the first 137 amino acids), which is more stable and suitable for *in vitro* assays (10).

**Figure 3:**
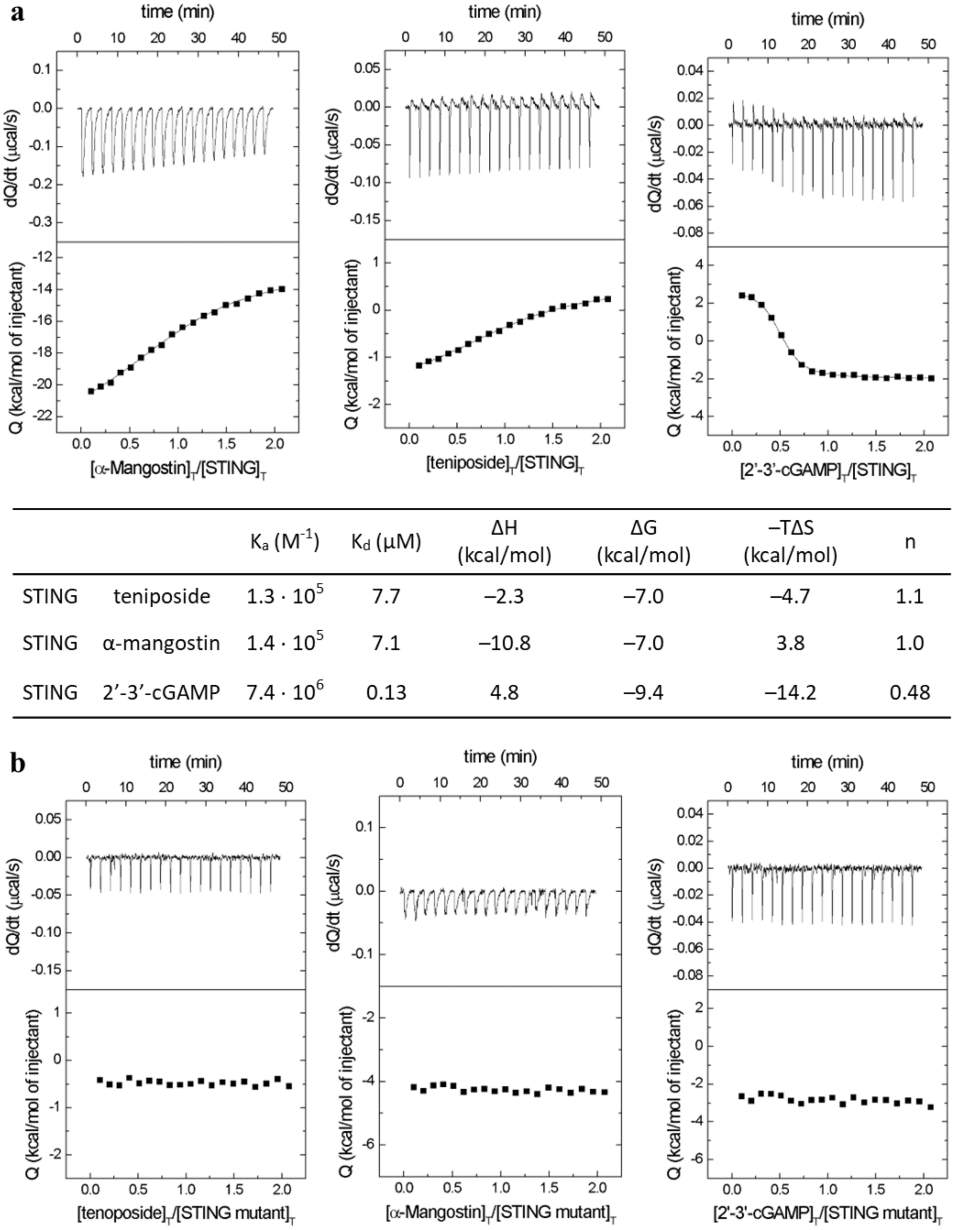
Isothermal titration calorimetry analysis of ligand binding to recombinant STING-LBD. **(a)** The top panels display heat flow rates over time for the binding of Teniposide, α-mangostin, and 2´3´-cGAMP to the STING ligand-binding domain (LBD), while the bottom panels show the total heat evolved per injection. The accompanying table summarizes the affinity constants, thermodynamic parameters, and stoichiometry values for each interaction. **(b)** Negative controls using a double mutant STING-LBD (R238A/Y240A), showing no detectable ligand binding. Experiments were conducted at 25°C in 20 mM HEPES, 150 mM KCl, pH 7.5. Recombinant STING-LBD (20 µM) was titrated with 300 µM of each ligand.

ITC analysis revealed that Teniposide binds STING-LBD exothermically (ΔH = -2.3 kcal/mol; **Figure 3**) with a 1:1 stoichiometry, where two Teniposide molecules bind a STING-LBD dimer (28). This binding profile is similar to α-mangostin (29) but distinct from cGAMP, which binds endothermically with a 1:2 stoichiometry (**Figure 3** (30)). Teniposide’s binding affinity is lower than that of cGAMP, indicating a weaker interaction under the tested conditions (**Figure 3a**).

To identify key residues involved in Teniposide binding, we used a STING-LBD double mutant (R238A/Y240A), where the targeted residues are critical for interactions with cyclic dinucleotides (CDNs). ITC analysis showed that none of the tested ligands (Teniposide, α-mangostin, or cGAMP) could establish a stable interaction with the mutant, confirming the essential roles of R238 and Y240 in ligand binding (**Figure 3b**). These results suggest that Teniposide shares a similar binding site with CDNs and highlights its unique interaction properties with STING.

### Evaluation of the dynamic stability of the binding of two Teniposide units to STING

Given that the calorimetry assays undoubtedly indicated that two Teniposide units interacted with a STING homodimer, we carried out a computational study to propose a possible binding mode involving the simultaneous binding of two Teniposide units within the binding pocket of a STING homodimer.

**Figure 4:**
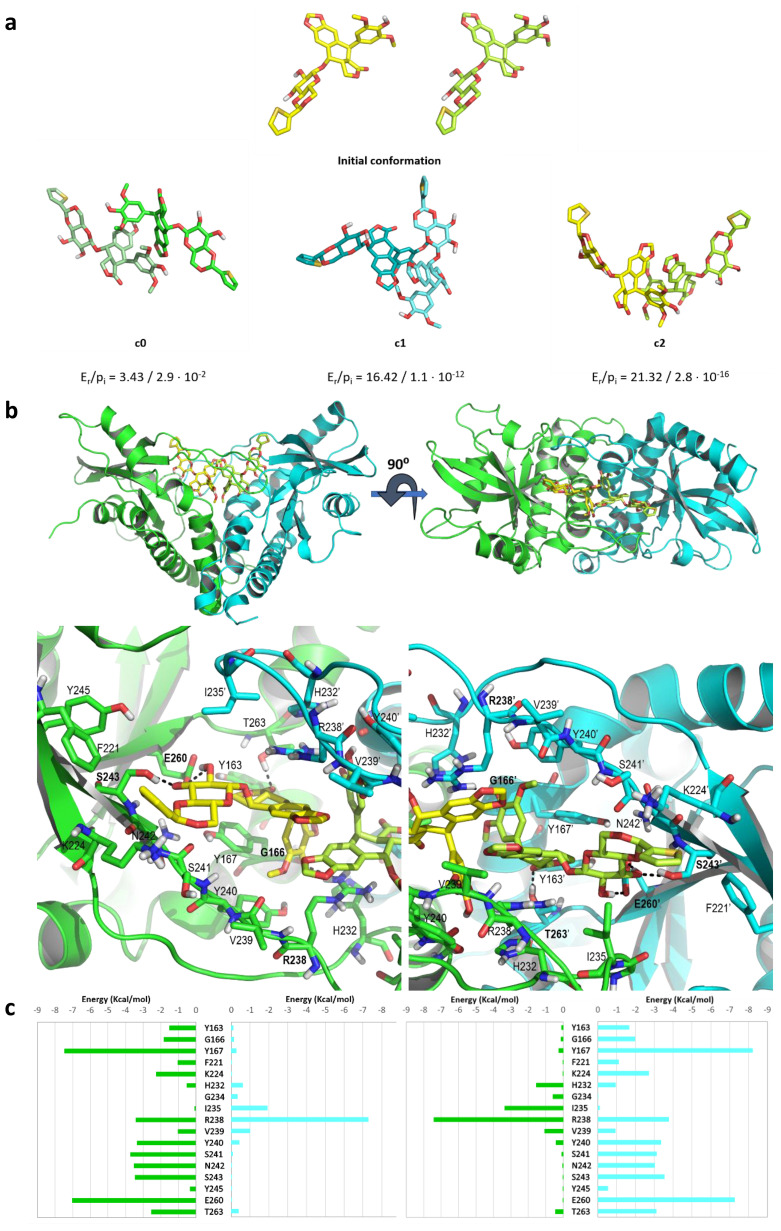
Computer model of the complex of STING with two units of Teniposide. **(a)** (top) Stick representation of the initial conformation, and (bottom) the representative structure of the first three most populated conformers extracted from the 100 ns MD simulation of the two Teniposide units in explicit water. The ratio between the relative energy of the conformer (E_r_) and the probability of each microstate according to the Boltzmann distribution (p_i_) are shown. **(b)** PyMOL stick and cartoon representation of the representative structure extracted from the 400 ns MD simulation of the STING homodimer (monomer A in green and monomer B in cyan) in complex with the untethered conformer c2 (Teniposide A and B represented as yellow and lime green sticks, respectively) seen both from the front (left) and from the top (right) to account for the symmetrical interaction. For the sake of clarity, in the detailed view of the ligand-receptor interactions (below), only polar hydrogens and the side chains of the amino acids that interact with the two Teniposide units are shown as sticks. The hydrogen bonds established between the ligands and the proteins are shown as black dashed lines and the amino acids involved in these interactions are labelled in bold. **(c)** Per residue energy decomposition, extracted from the MD simulation, of the interaction of both Teniposide units with the amino acids highlighted in **b** from monomers A and B (green and cyan bars, respectively).

To achieve this goal, we performed a 100 ns MD simulation of two Teniposide units in water and extracted the most populated conformers to obtain a rationale on how the two molecules would interact with one another (**Supplementary Figure S2**). The two molecules in the three most populated conformers (**Figure 4a**) were then manually tethered with alkyl chains to be able to treat them as one single molecule for docking purposes (**Supplementary Figure S3**). The structural information available prompted us to use the closed (PDB codes 4LOI (15) and 8A2I (31)); semi-open (PDB codes 6DXL and 6DXG (22)); and open conformations (PDB code 6MXE (23)) of STING as a protein target for the docking studies. To avoid a possible bias in the obtained binding mode brought about by the modeled loops in the absence of ligands, the docking was carried out without modeling the missing amino acids in the semi-open and open conformations. Of the three above-mentioned tethered conformers, only conformer c2 (**Figure 4a**) rendered a plausible binding mode to the 6MXE structure. In this binding mode, only one of the tethered Teniposide molecules (from now on referred to as Teniposide A) interacts with one of the STING monomers (from now on referred to as monomer A). Teniposide A establishes hydrogen bonds with S162, E260, and T267; a parallel-displaced π-π stacking interaction with the side chain of Y167, a cation-π interaction with K224 and multiple van der Waals contacts with residues Y163, F221, S243 and Y245 (**Supplementary Figure S4**). Taking into account that the binding of Teniposide induces STING activity, we rationalized that the binding should be either in the closed or at least the semi-open conformation. The main issue was that the crystallographic structures 6DXL and 6DXG miss quite large segments of the loops that should form the β-sheet lid structure over the CDN-binding site, and the modeling of these loops would introduce much uncertainty to the model. We searched for other alternatives and selected 8A2I (31). In this crystal structure, STING is bound to a CDN analog that has three aromatic rings substituted at position 7 of the adenine. This modifies the typically closed geometry of the β-sheet lid and impairs the resolution of I235 in one of the sheets. To propose a binding mode with the same STING variant as the one used in the ITC experimental analysis, the R232H mutation was manually introduced on the 8A2I structure. Upon superimposition of monomer A of the selected docking model for the tethered conformer c2 bound to 6MXE to the R232H mutated 8A2I, we found that albeit there were steric clashes between the amino acids of the β-sheet lid and the two Teniposide units, those could be reduced by successive minimizations. For those minimizations and further computational treatment, the Teniposide units were untethered to allow them to freely adjust to the CDN-binding site. Once the structure was optimized (**Supplementary Figure S5**), a 400 ns MD simulation was carried out to evaluate the stability of the computer-generated model system, analyze its dynamic behavior, and study the ligand-receptor interactions that could account for the previously described experimental results.

The starting structure of this MD simulation had Teniposide A establishing distinct interactions with monomer A, whilst Teniposide B was not fully accommodated in the CDN-binding site (**Supplementary Figure S5**). Therefore, the binding of Teniposide A was stable throughout the MD simulation, whereas Teniposide B was stabilized after the initial 110 ns of the simulation and remained stable until the end (**Supplementary Figure S6**). Interestingly, the initially close Teniposide units were slightly separated so that each one could fully interact with its corresponding STING monomer (**Figure 4b**). The adjustment of Teniposide B rendered a symmetrical binding mode of the two Teniposide units to the STING homodimer (**Figure 4b**) similar to those already described in the literature (15, 22, 23, 32). Once fully adjusted, the Teniposide units establish van der Waals contacts between the [1,3] dioxol ring of the polycyclic ring system.

The per-residue energy decomposition extracted from the entire simulation shows a clear symmetrical interaction of the two Teniposide units where each one establishes major interactions with its corresponding STING monomer (Monomer A, in green, with Teniposide A, in yellow, and monomer B, in cyan with Teniposide B, in lime green, as depicted in **Figure 4c**). In the proposed binding mode, each Teniposide is stabilized by the hydrogen bonds and van der Waals interactions established with amino acids of their corresponding subunit. The pendant ring is stabilized by a hydrogen bond with the backbone of G166 and the side chain of R238; the polycyclic ring system with the side chain of T263; and the glycosidic moiety with the side chain of S243 and E260. A very strong interaction with Y167 complemented with that of Y240 further stabilized the position of the pendant ring. Additionally, each Teniposide unit is stabilized by a strong cation-π interaction with the side chain of R238 of the opposite monomer. This strong interaction with R238 along with the above-mentioned interaction with Y167 are likely responsible for maintaining the semi-open conformation of STING and the amino acids that form the lid in place throughout the simulation time. These interactions are also present in the binding of cGAMP to STING as found in crystallized structures 4LOI and 7SII (15, 33), and in the crystal structures of the modified CDN bound to STING (8A2I) (31). Furthermore, considering the binding mode and the per-residue energy decomposition, these results are in line with the lack of binding of Teniposide to the double mutation at residues R238A and Y240A.

## Discussion

In the present work, we describe the discovery, binding, and functional characterization of Teniposide as a potential direct STING agonist ligand. Teniposide was first identified as a putative STING ligand through a HTVS. Here we have used available structural data of STING to further explore and try to understand the structural basis of the activation of the protein as well as to test Teniposide as a potential STING-direct activation candidate.

Teniposide is known to inhibit the Topoisomerase II enzyme, leading to DNA breaks. Fragments of DNA released into the cytoplasm can be sensed by cGAS, which in turn produces cGAMP, the natural ligand responsible for activating STING (26). Here we present that the *IFNB1* expression was exclusively achieved in WT THP1 cells but not in STING KO cells, indicating a STING dependence for Teniposide activation of IFN-β induction pathway. Moreover, cGAS knockout (KO) cells still respond to Teniposide treatment, suggesting the potential of Teniposide to engage STING through a cGAS-cGAMP-independent mechanism, possibly through direct action. Further examination indicates that IFI16 is dispensable to stimulate the IFN-β induction pathway in response to Teniposide in THP1 cells, particularly evident early point as 4 hpt **Figure 1**). At this point *IFNB1* expression is elevated in cells treated with Teniposide compared to those treated with cGAMP alone. This increase could potentially result from a synergistic effect of directly activating STING and delaying the production of cGAMP molecules by cGAS following DNA breakage. Direct STING activation by Teniposide could then be a potential mechanism in addition to a cGAS-dependent pathway. The proposed model of STING stimulation is depicted in **Supplementary Figure S7A**. The direct binding model is supported by the observation that *IFNB1* expression is enhanced upon cGAMP stimulation in cells pretreated with IFN−β, but that effect is not observed in response to Teniposide in IFN−pretreated cells. This difference can be explained by the fact that cGAS, codified by the C6orf150 gene is an interferon−inducible (https://www.omim.org/entry/613973). In this context, elevated cGAS, or other cGAMP interacting factor, levels could sensitize cells to respond more robustly to cGAMP, whereas Teniposide, which may activate STING independently of cGAMP production, is unaffected by changes in cGAS expression at early time points (**Figures 2d, e**).

Although other DNA sensors such as ZBP1 or DDX41 have been implicated in triggering the STING pathway, ITC analysis provides *in vitro* evidence that supports a direct interaction between two Teniposide units and STING. Teniposide binding to STING appears to be specific, as structurally related compounds such as etoposide fail to induce and to bind (**Supplementary Figure S7C**) and stimulate STING related IFN-β induction (26).

The predicted computer model is also in line with the Teniposide-induced activation of STING, as the binding allows a closed conformation of the lid on top of the CDN-binding site albeit not in a fully formed β-sheet lid. This occurrence can be found in the symmetrical binding of the STING agonists in PDB codes 6DXG, 6DXL and 8TG6, as well as in that of PDB code 8A2I.

Moreover, the computer model is in line with the experimental results obtained by ITC for the double mutant at residues R238A and Y240A. The observed loss of binding in the case of the double mutant that completely abolishes the ability to respond to Teniposide is in line with the energetic interactions established with the side chains of R238 and Y240 found in the per-residue energy decomposition. The loss of these interactions clearly accounts for the lack of binding affinity in the double mutants.

The occurrence of two identical ligand molecules binding the homodimer is not uncommon. Several crystal structures have been deposited in the PDB involving mouse and human STING in the open, closed and/or semi-open conformations (14, 15). Visual inspection of STING crystal structures proved that the occurrence of two identical molecules symmetrically binding to the STING homodimer is not uncommon, as there are several examples of a symmetrical binding in both mouse STING (DMXAA (1YE) in 4LOL) (15) and human STING (activator Compound 1 (HGJ) in 6DXG (22), and inhibitors Compound 18 (K5S) and K5P in 6MXE and 6MX3, respectively (23)). This has led to the synthesis of the structurally related tethered STING-activating compounds like Compound 2 (HG4) in 6DXL (22) and the current on clinical development Compound 3 (renamed HB3089), in 8GT6 (32).

It is important to highlight also that THP-1 cells carry the HAQ allele of STING. Despite this allele, STING is retaining the capacity to respond both to cGAMP and Teniposide (34). The plasmids used to overexpress human STING and the recombinant protein used for ITC present to Wt allele (RGR). Both, the RGR and HAQ alleles could be considered to determine differences in the STING protein activity will respond to Teniposide. These differences in the affinity and the differential ability to induce *IFNB1* expression and downstream effects can be determined in future experiments.

Teniposide, marketed as Vumon^®^ by BRISTOL-MYERS SQUIBB, is a medication utilized in the treatment of cancer patients (13). Based on our data presented here, it is anticipated that its therapeutic effects may stem from various stimuli, including direct activation of STING described here, indirect cGAS stimulation (26) and bystander trans stimulation by dead cells reported by the group of Glenn Barber (35). The selectivity index of Teniposide in mediating *IFNB1* gene expression is 3,27 (**Supplementary Figure S10**), indicating a favorable balance between pathway activation and cytotoxicity under the tested conditions. Additionally, the efficacy of these effects could vary depending on the characteristics of different STING polymorphisms. Patients with reduced or absent activation of STING via cGAS-cGAMP-dependent stimulation might experience diminished immune stimulation, whereas those with STING competence would likely respond favorably to Teniposide in such circumstances. Consequently, besides the ability of Teniposide to inhibit Topoisomerase II and perform its well-described antitumoral effects, we consider this molecule to be a promising candidate for immunotherapy, acting as a modulator of IFN-β signaling and an inducer of STING-related innate immune responses. Development of potential Teniposide derivatives or Teniposide analogues could improve the described STING activation described here and be used to complement the oncolytic with the immunostimulatory potential of some antitumoral drugs.

### Study limitations

Additional biophysical assays such as surface plasmon resonance (SPR) could be employed to improve the characterization of the binding kinetics and cellular thermal shift assay (CETSA) to assess STING-Teniposide interaction in a more physiological setting.

Additional characterization of STING downstream signaling kinetics, including STING dimerization, TBK1 phosphorylation, IRF-3 or p65 (NK-κB subunit) phosphorylation, dimerization, or cytoplasmic to nuclear translocation, could improve the level of detail in the activation of the pathway. The little window of activation to discriminate direct binding from indirect cGAS stimulation limits a better description.

The binding mode of Teniposide suggests interactions with STING residues similar to those engaged by cGAMP (**Supplementary Figure S8**). However, demonstrating a distinct binding mode remains challenging, as it is difficult to identify specific residues that are uniquely critical for Teniposide binding without also affecting cGAMP interaction. This overlap complicates efforts to clearly distinguish the binding mechanisms of the two ligands.

Experiments to detect IFN-β cytokine in the supernatant of Teniposide-treated cells have been performed. Although this IFN can be detected, it has not been possible to discriminate the IFN directly induced by Teniposide binding to STING and the one that results from Teniposide´s action on topoisomerase II that results in free DNA that can trigger cGAS (as described by Wang et al. (26)) or IFI16. The experiments performed with cGAS KO or IFI16 KO THP1 cells to exclude this second possibility resulted in a cellular toxicity that inhibited the cells from producing IFN-β fast enough to be detected in enough amounts (data not shown). The use of other cGAS knockout cell lines may reveal differences in Teniposide sensitivity, including variations in the therapeutic window and toxicity. Future experiments should address this issue to better characterize and distinguish the mechanisms underlying Teniposide-induced STING activation. We have performed an MTS assay to estimate cell viability (**Supplementary Figure S9**). It can be observed that the doses of Teniposide used compromise cell viability in our THP1 cellular model.

## Data Availability

The original contributions presented in the study are included in the article/**Supplementary Material**. Further inquiries can be directed to the corresponding authors.
