# Supplementary material for: STING activation by teniposide: a potential direct mechanism beyond cGAS stimulation
Source: Front Immunol. 2026 Jan 2;16:1677836. doi: 10.3389/fimmu.2025.1677836 (PMC12808447; doi:10.3389/fimmu.2025.1677836)
Supplement: Supplementary file 1 [file DataSheet1.pdf]

Supplementary Figure 1: ***Top.*** PyMOL surface representation of the predicted binding mode of one Teniposide unit to STING in its open conformation (PDB code 4F5W) extracted from HTVS- ***Bottom.*** Docking scores from HTVS for the natural ligand cGAMP in the open and closed conformations, and Teniposide in the open conformation.

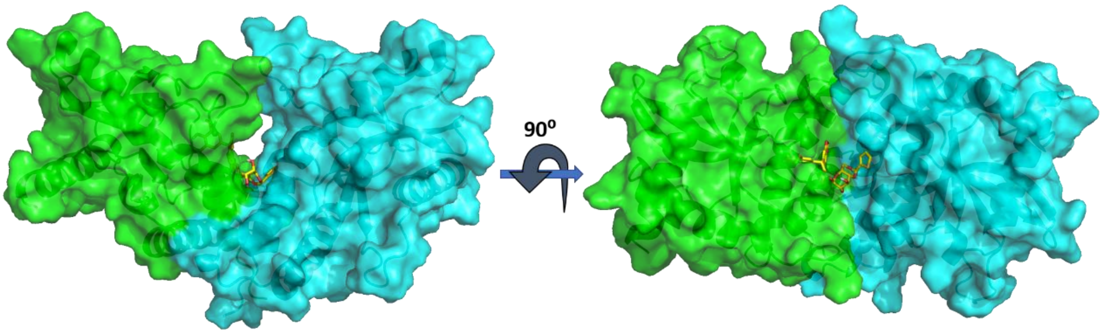

PDB	Conformation	Ligand	Docking score (Kcal/mol)
4LOI	closed	cGAMP	-11.096
4F5W	open	cGAMP	-5.168
4F5W	open	Teniposide	-7.047
