# Supplementary material for: STING activation by teniposide: a potential direct mechanism beyond cGAS stimulation
Source: Front Immunol. 2026 Jan 2;16:1677836. doi: 10.3389/fimmu.2025.1677836 (PMC12808447; doi:10.3389/fimmu.2025.1677836)
Supplement: Supplementary file 2 [file DataSheet2.pdf]

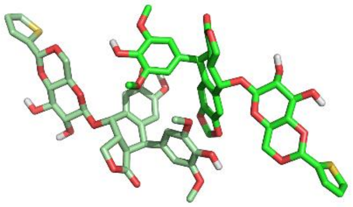

c0
$E_r/p_i = 3.43 / 2.9 \cdot 10^{-2}$

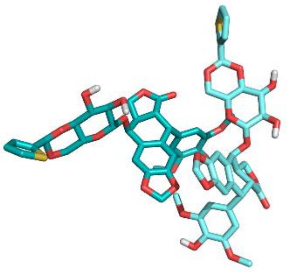

c1
$E_r/p_i = 16.42 / 1.1 \cdot 10^{-12}$

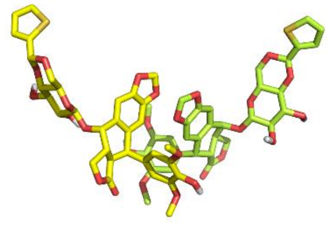

c2
$E_r/p_i = 21.32 / 2.8 \cdot 10^{-16}$

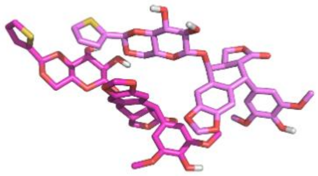

c3
$E_r/p_i = 0 / 0.79$

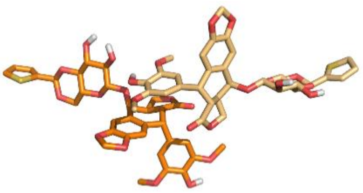

c4
$E_r/p_i = 0.89 / 0.16$

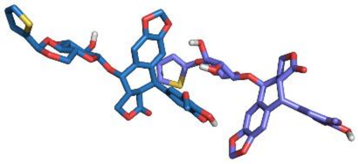

c5
$E_r/p_i = 10.06 / 2.8 \cdot 10^{-8}$

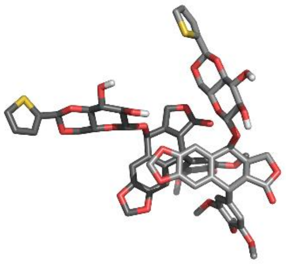

c6
$E_r/p_i = 7.61 / 1.6 \cdot 10^{-6}$

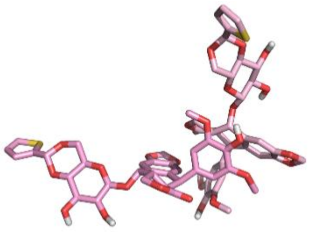

c7
$E_r/p_i = 13.42 / 4.5 \cdot 10^{-11}$

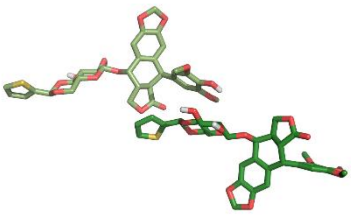

c8
$E_r/p_i = 1.51 / 1.2 \cdot 10^{-2}$

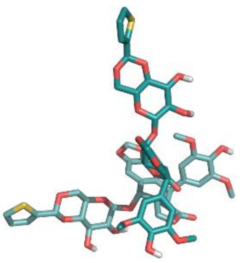

c9
$E_r/p_i = 14.73 / 1.8 \cdot 10^{-12}$

Supplementary Figure 2: Stick-model view of the representative structure of the ten conformers extracted from the 100 ns MD simulation of the two Teniposide molecules in explicit water. The ratio between the relative energy of the conformer (E_r) and the probability of each microstate according to the Boltzmann distribution (p_i) are shown.
