# Supplementary material for: STING activation by teniposide: a potential direct mechanism beyond cGAS stimulation
Source: Front Immunol. 2026 Jan 2;16:1677836. doi: 10.3389/fimmu.2025.1677836 (PMC12808447; doi:10.3389/fimmu.2025.1677836)
Supplement: Supplementary file 3 [file DataSheet3.pdf]

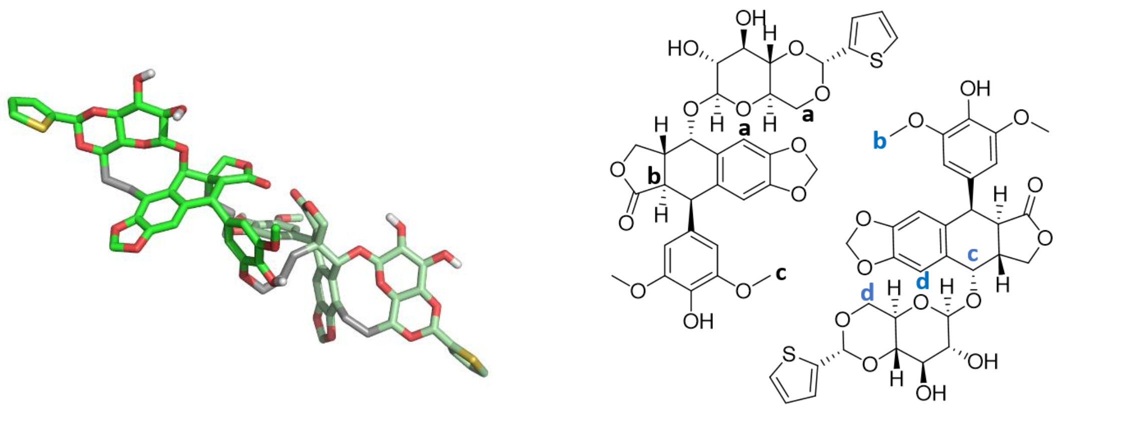

c0

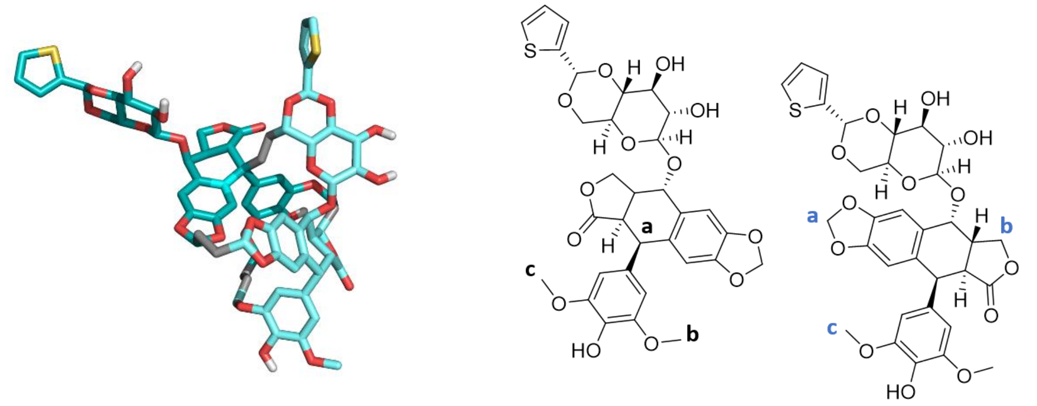

c1

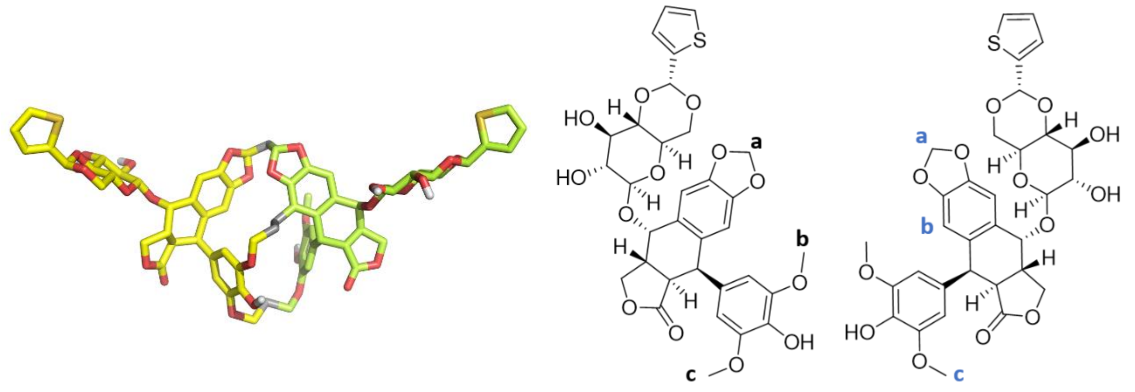

c2

Supplementary Figure 3: Stick-model view of the representative structure of the three most populated conformers of the two Teniposide molecules in water solvent tethered for docking purposes. The alkyl chains used to maintain the interaction between the two monomers within the conformer are shown in grey sticks. The corresponding attaching points of the alkyl chains are identified by letters **a** to **e** and color-coded for each monomer.
