# Supplementary material for: STING activation by teniposide: a potential direct mechanism beyond cGAS stimulation
Source: Front Immunol. 2026 Jan 2;16:1677836. doi: 10.3389/fimmu.2025.1677836 (PMC12808447; doi:10.3389/fimmu.2025.1677836)
Supplement: Supplementary file 5 [file DataSheet5.pdf]

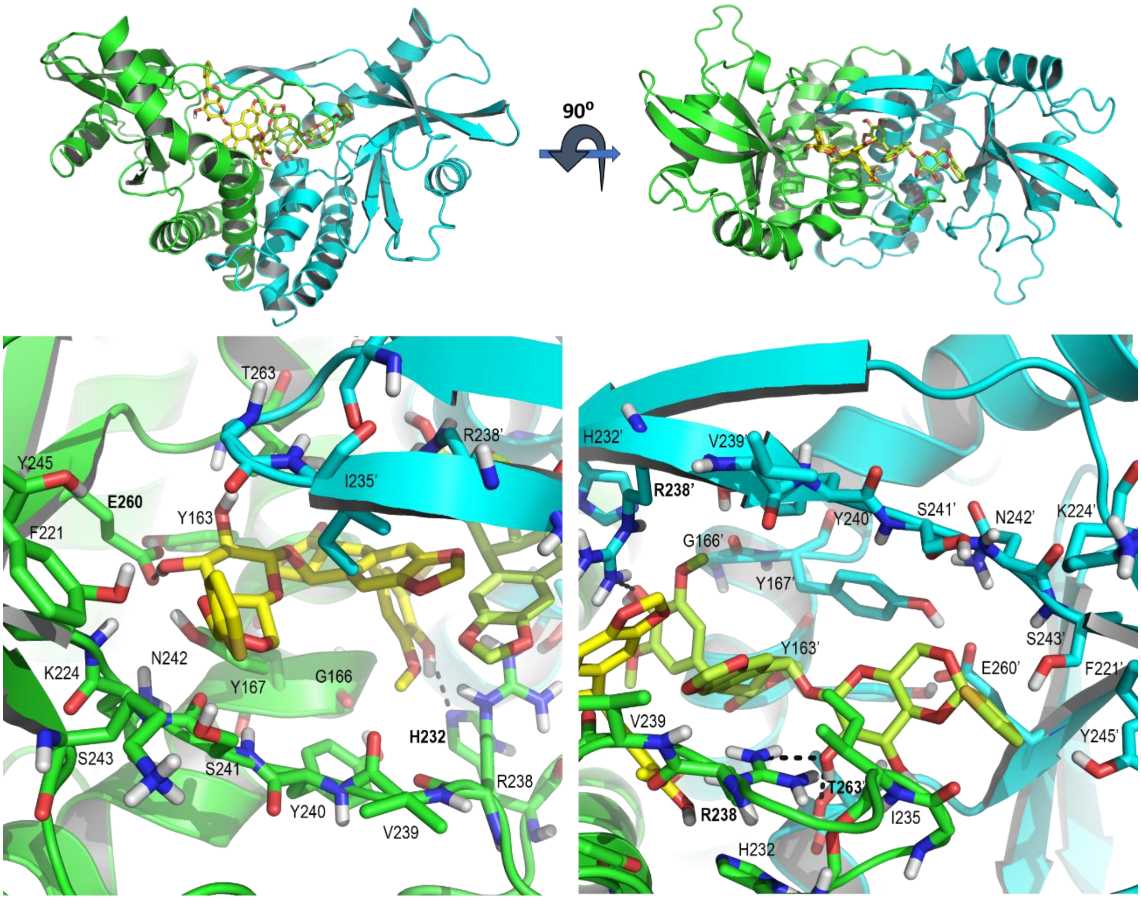

Supplementary Figure 5: PyMOL stick and cartoon representation of the untethered minimized structure of the two Teniposide molecules bound to the STING homodimer (PDB code 8A2I) used for the 400 ns MD simulation. ***Top:*** Overall view of the initial complex: Teniposide A (yellow) interacts with monomer A (green) of the STING dimer and Teniposide B (bright green) is oriented towards monomer B (cyan) of the STING dimer. ***Bottom:*** detail of the interaction of the binding mode of Teniposide A (left) and Teniposide B (right). For the sake of clarity, only polar hydrogens and the side chains of the amino acids that interact with the Teniposide molecules are shown as sticks. The hydrogen bonds established between the ligands and the proteins are shown as black dashed lines and the amino acids involved in these interactions are labelled in bold.
