# Supplementary material for: STING activation by teniposide: a potential direct mechanism beyond cGAS stimulation
Source: Front Immunol. 2026 Jan 2;16:1677836. doi: 10.3389/fimmu.2025.1677836 (PMC12808447; doi:10.3389/fimmu.2025.1677836)
Supplement: Supplementary file 6 [file DataSheet6.pdf]

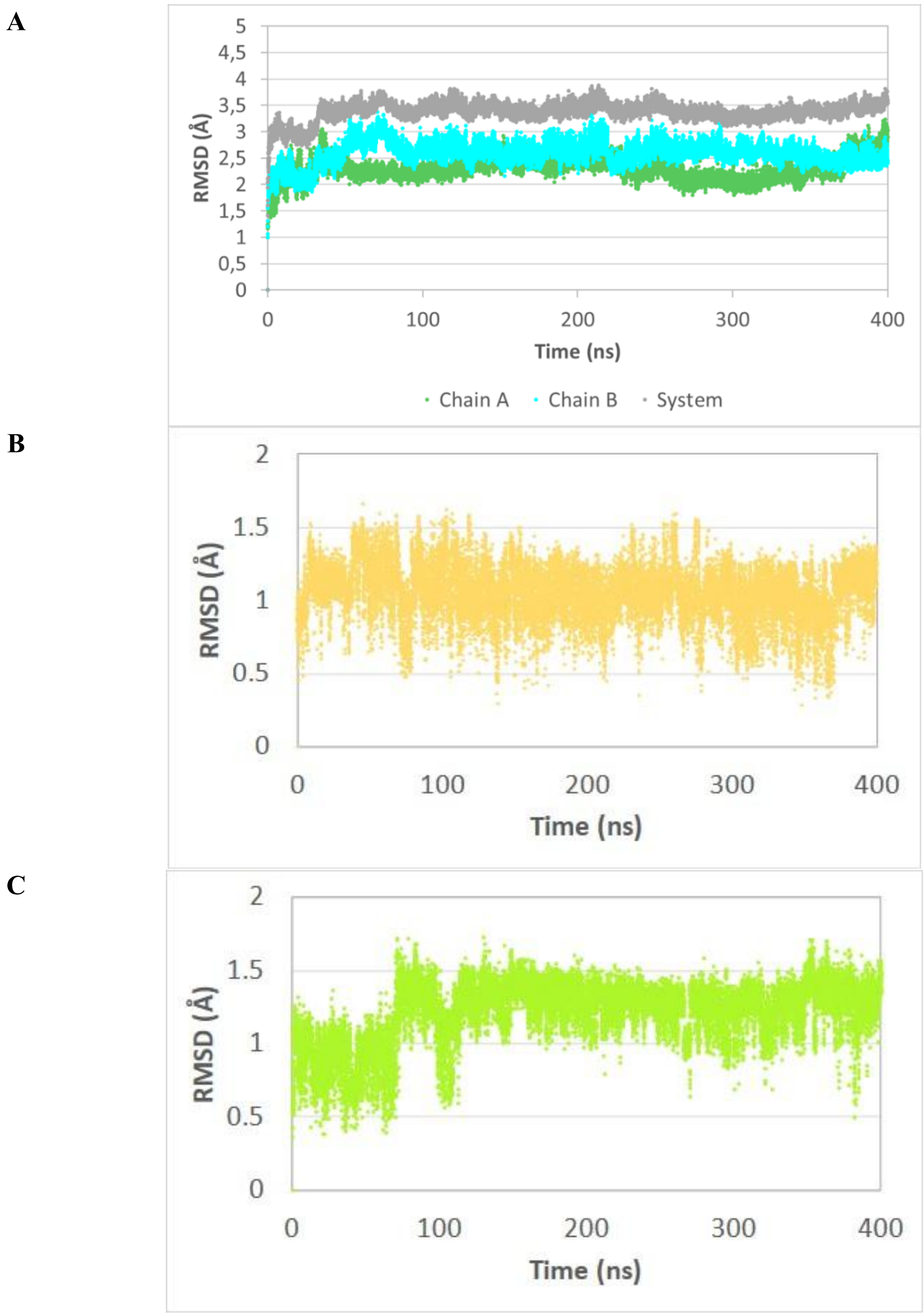

Supplementary Figure 6: RMSD (Å) analysis of the 400 ns MD simulation of the binding mode of the two Teniposide molecules to the STING homodimer where the first step of the MD simulation was used as reference. **A**. Comparative RMSD plot that includes the RMSD values from backbone of monomer A (green), monomer B (cyan), and the whole complex including the two Teniposide molecules (grey). **B**. RMSD plot of the fluctuation of the Teniposide A molecule (yellow). **C**. RMSD plot of the fluctuation of the Teniposide B molecule (bright green).
