# Supplementary material for: STING activation by teniposide: a potential direct mechanism beyond cGAS stimulation
Source: Front Immunol. 2026 Jan 2;16:1677836. doi: 10.3389/fimmu.2025.1677836 (PMC12808447; doi:10.3389/fimmu.2025.1677836)
Supplement: Supplementary file 7 [file DataSheet7.pdf]

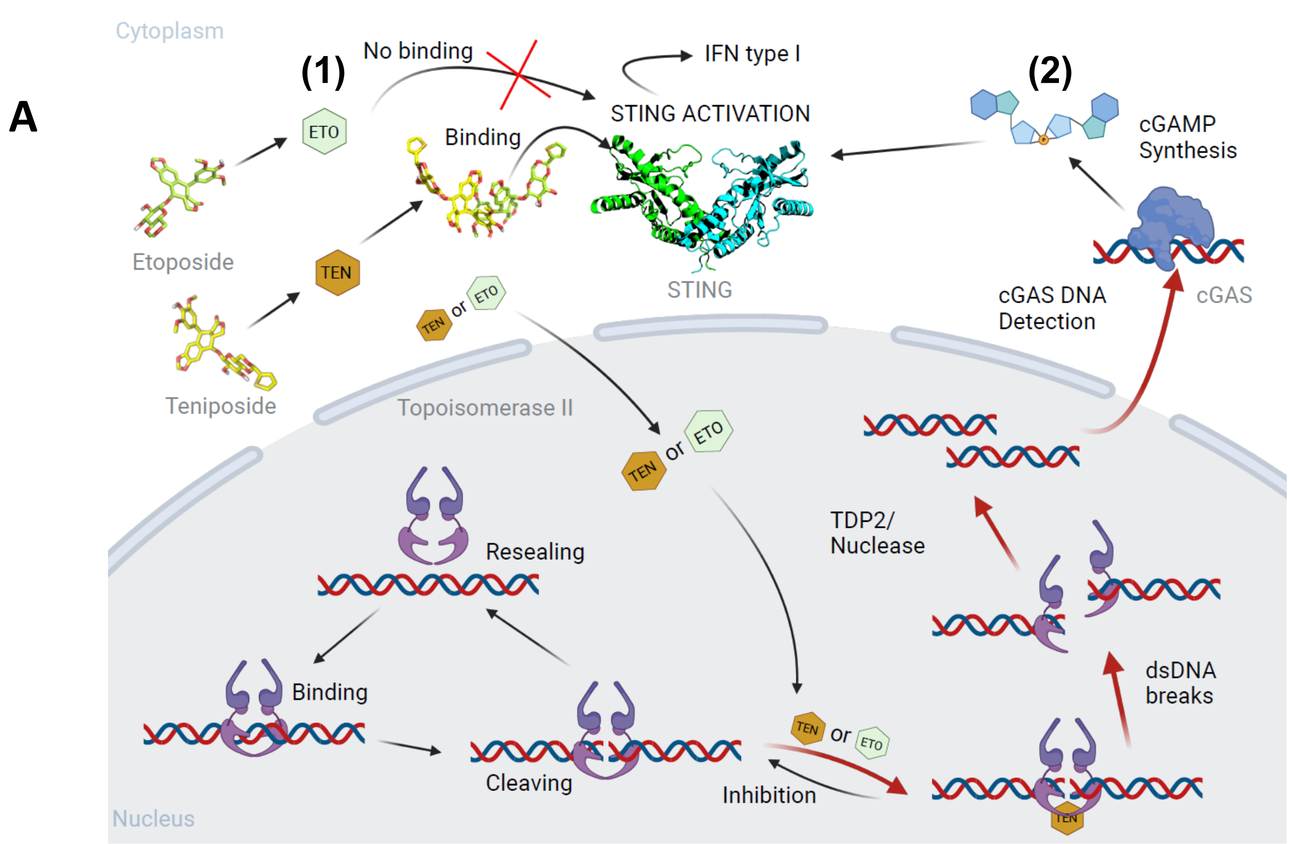

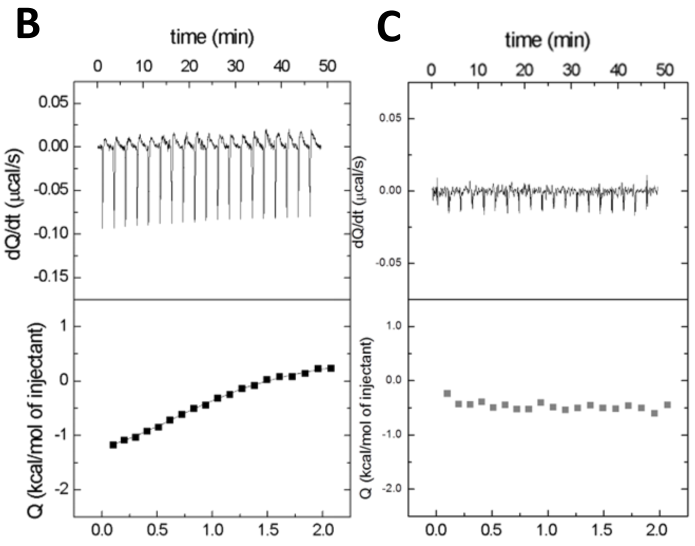

Supplementary Figure 7: Teniposide proposed mechanism of STING induction:

A) Schematic representation of Teniposide-mediated activation of STING through two potential mechanisms: (1) direct binding to STING, or (2) indirect activation via topoisomerase II inhibition, leading to double-stranded DNA (dsDNA) breaks. These breaks can activate the dsDNA sensor cGAS, which in turn produces cGAMP, a secondary messenger that stimulates STING activation. Etoposide instead can only trigger the second mechanism. Wang et al. (26) compare the antitumor properties of Teniposide and Etoposide, both of which are Topoisomerase II inhibitors. The results demonstrate that Teniposide exhibits a higher adjuvant antitumor effect compared to Etoposide. **B)** The top panels display heat flow rates over time for the binding of Teniposide (left) and **C)** Etoposide (right) to the STING ligand-binding domain (LBD). The bottom panels illustrate the total heat released per injection. Experiments were performed at 25°C in a buffer containing 20 mM HEPES, 150 mM KCl, pH 7.5. Recombinant STING-LBD (20 µM) was titrated with 300 µM of each ligand.
