# Supplementary material for: STING activation by teniposide: a potential direct mechanism beyond cGAS stimulation
Source: Front Immunol. 2026 Jan 2;16:1677836. doi: 10.3389/fimmu.2025.1677836 (PMC12808447; doi:10.3389/fimmu.2025.1677836)
Supplement: Supplementary file 8 [file DataSheet8.pdf]

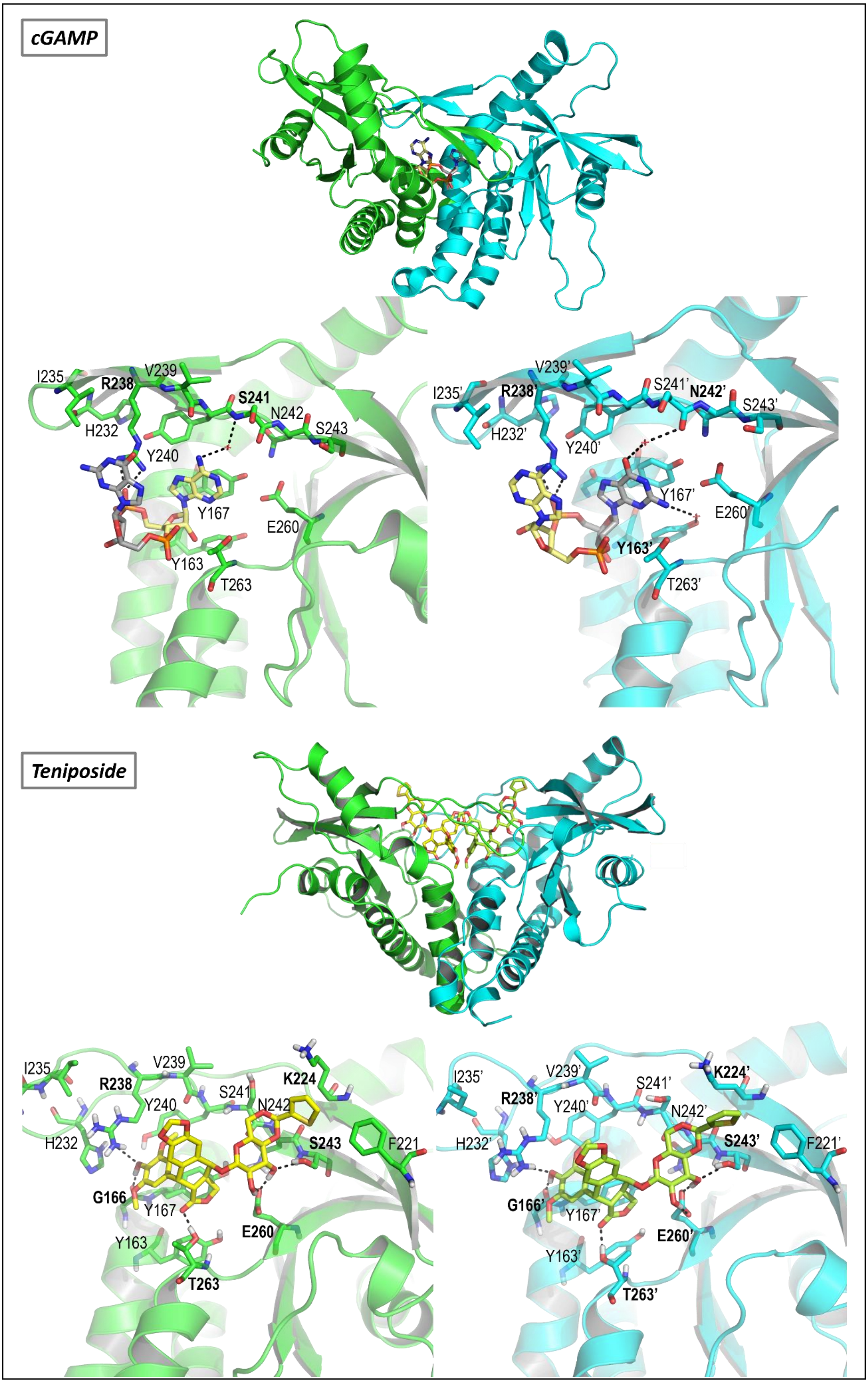

cGAMP
I235
R238
V239
S241
N242
S243
H232
Y240
Y167
E260
Y163
T263
I235'
R238'
V239'
S241'
N242'
S243'
H232'
Y240'
Y167'
E260'
Y163'
T263'
Teniposide
K224
F221
G166
K224'
F221'
G166'

Supplementary Figure 8. Comparison of the binding modes of cGAMP (PDB code 4LOH, *up*) and Teniposide (proposed model, *bottom*) to STING homodimer. Chain A, Chain B, Teniposide A and B are colored in green, cyan, yellow and bright green, respectively. For clarity, only the residues involved in the binding mode of cGAMP and Teniposide are represented as sticks, with the residues belonging to Chain B labeled with a prime symbol. Hydrogen bonds are indicated as black dashes and water molecules as red crosses.
