# Supplementary material for: STING activation by teniposide: a potential direct mechanism beyond cGAS stimulation
Source: Front Immunol. 2026 Jan 2;16:1677836. doi: 10.3389/fimmu.2025.1677836 (PMC12808447; doi:10.3389/fimmu.2025.1677836)
Supplement: Supplementary file 9 [file DataSheet9.pdf]

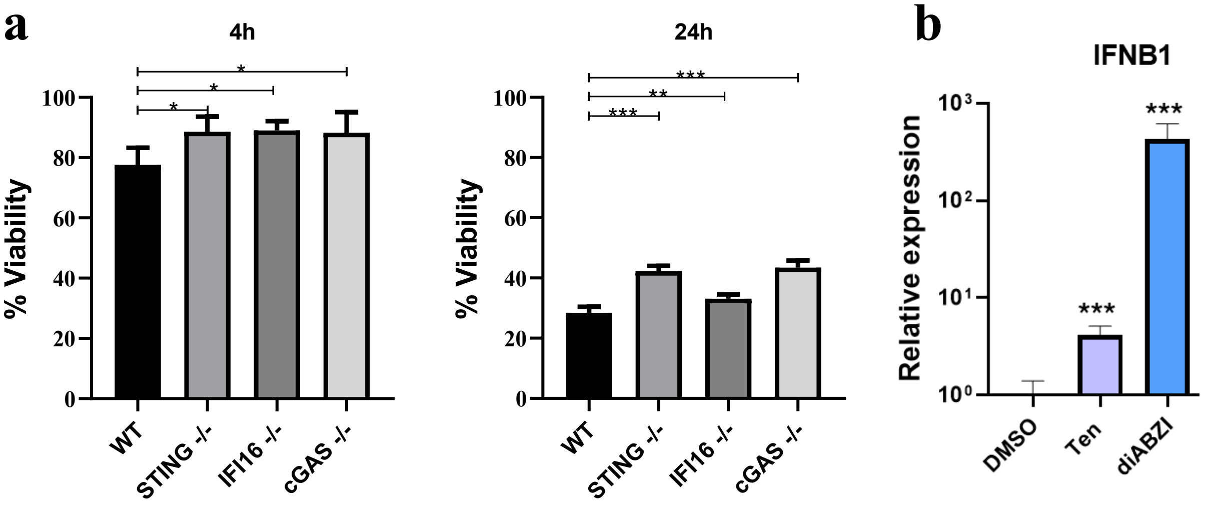

Supplementary Figure 9: Teniposide MTS viability assay in THP1 cells and ability to trigger IFNB1 expression in MAVS KO cells :

a) 10,000 cells/well in a 96-well plate (WT, STING KO, IFI16 KO, cGAS KO) were treated with 3 µM Teniposide or DMSO (control) to each well. 4 and 24 hours post-treatment MTS Assay cell viability is performed. Cell viability is represented as a percentage relative to DMSO-treated controls. b) MAVS KO THP1 cells treated for 8h with DMSO, 3µM Teniposide, or 1µM diABZI.
