# Supplementary material for: STING activation by teniposide: a potential direct mechanism beyond cGAS stimulation
Source: Front Immunol. 2026 Jan 2;16:1677836. doi: 10.3389/fimmu.2025.1677836 (PMC12808447; doi:10.3389/fimmu.2025.1677836)
Supplement: Supplementary file 10 [file DataSheet10.pdf]

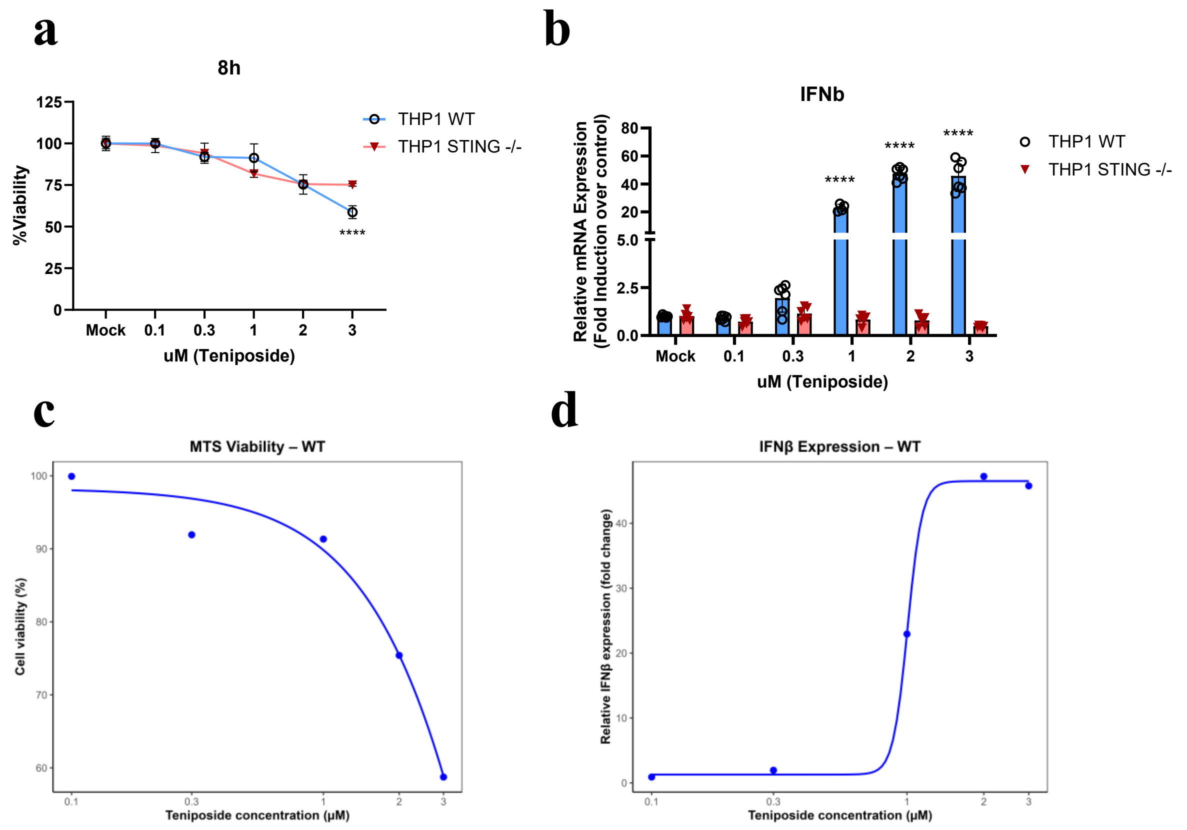

d

Teniposide – CC50, EC50 and Therapeutic Index (TI)

cell_line	CC50	EC50	Therapeutic_Index
WT	3.290	1.010	3.270

Supplementary Figure 10: Selectivity index of Teniposide in THP1 cells 8 hours post treatment: (a, b) 20,000 cells/well in a 96-well plate (WT, and STING KO) were treated with different concentrations of Teniposide or DMSO (control) to each well. 8 hours post-treatment a cell viability assay (MTS) and a gene expression analysis (RT-qPCR) was performed. Cell viability is represented as a percentage relative to DMSO-treated controls and *IFNB1* expression is normalized to DMSO-treated controls. **(c, d)** Correlation analysis between cytotoxicity and *IFNB1* induction by teniposide. Scatter plots show the relationship between cell viability and *IFNB1* expression levels. **(e)** Table representing cytotoxic concentration 50 (CC50), effective concentration 50 (EC50), and therapeutic index (CC50/EC50).
