# Supplementary material for: STING activation by teniposide: a potential direct mechanism beyond cGAS stimulation
Source: Front Immunol. 2026 Jan 2;16:1677836. doi: 10.3389/fimmu.2025.1677836 (PMC12808447; doi:10.3389/fimmu.2025.1677836)
Supplement: Supplementary file 11 [file Table1.docx]

**Supplementary Table 1:**

| **RTqPCR primers** | |  |  |
| --- | --- | --- | --- |
| **Gene** |  | **Sequence** | **Organism** |
| ***18S*** | FW | GGCCCTGTAATTGGAATGAGTC | *Homo sapiens* |
|  | RV | CCAAGATCCAACTACGAGCTT |  |
| ***ACTB*** | FW | AGGCACCAGGGCGTGAT | *Homo sapiens* |
|  | RV | GCCCACATAGGAATCCTTCTGAC |  |
| ***IFNB1*** | FW | TCTGGCACAACAGGTAGTAGGC | *Homo sapiens* |
|  | RV | GAGAAGCACAACAGGAGAGCAA |  |
| ***IL6*** | FW | AGAGGCACTGGCAGAAAACAAC | *Homo sapiens* |
|  | RV | AGGCAAGTCTCCTCATTGAATCC |  |
| ***MXA*** | FW | GTTTCCGAAGTGGACATCGCA | *Homo sapiens* |
|  | RV | GAAGGGCAACTCCTGACAGT |  |
| ***18s*** | FW | ATGCTCTTAGCTGAGGTGCCCG | *Mus musculus* |
|  | RV | ATTCCTAGCTGCGGTATCCAGG |  |
| ***Actb*** | FW | CGCGTCCACCCGCGAG | *Mus musculus* |
|  | RV | CCTGGTGCCTAGGGCG |  |
| ***Ifnb1*** | FW | CAGCTCCAAGAAAGGACGAAC | *Mus musculus* |
|  | RV | GGCAGTGTAACTCTTCTGCAT |  |
| ***Il6*** | FW | TGAGATCTACTCGGCAAACCTAGTG | *Mus musculus* |
|  | RV | CTTCGTAGAGAACAACATAAGTCAGATACC |  |
| ***Mx1*** | FW | GACCATAGGGGTCTTGACCAA | *Mus musculus* |
|  | RV | AGACTTGCTCTTTCTGAAAAGCC |  |
